# Non-Host Plant Volatiles Disrupt Sex Pheromone Communication in a Specialist Herbivore

**DOI:** 10.1038/srep32666

**Published:** 2016-09-02

**Authors:** Fumin Wang, Jianyu Deng, Coby Schal, Yonggen Lou, Guoxin Zhou, Bingbing Ye, Xiaohui Yin, Zhihong Xu, Lize Shen

**Affiliations:** 1 Department of Plant Protection, School of Agriculture and Food Science, Zhejiang Agriculture and Forestry University, Lin’an, Hangzhou, Zhejiang, 311300, China; 2 Department of Entomology and W. M. Keck Center for Behavioral Biology, North Carolina State University, Campus Box 7613, Raleigh, NC 27695–7613, USA; 3 National Key Laboratory of Rice Biology, Institute of Insect Science, Zhejiang University, Hangzhou, Zhejiang, 310058, China

## Abstract

The ecological effects of plant volatiles on herbivores are manifold. Little is known, however, about the impacts of non-host plant volatiles on intersexual pheromonal communication in specialist herbivores. We tested the effects of several prominent constitutive terpenoids released by conifers and *Eucalyptus* trees on electrophysiological and behavioral responses of an oligophagous species, *Plutella xylostella*, which feeds on Brassicaceae. The non-host plant volatile terpenoids adversely affected the calling behavior (pheromone emission) of adult females, and the orientation responses of adult males to sex pheromone were also significantly inhibited by these terpenoids in a wind tunnel and in the field. We suggest that disruption of both pheromone emission and orientation to sex pheromone may explain, at least in part, an observed reduction in herbivore attack in polyculture compared with monoculture plantings. We also propose that mating disruption of both male and female moths with non-host plant volatiles may be a promising alternative pest management strategy.

Co-evolution among herbivores and plants shapes communities of specialist and generalist feeders. Herbivores use plant traits – including color, shape, texture and chemical composition – to orient to plants and accept or reject them as substrates for feeding and oviposition. Insects use visual and olfactory cues to assess habitat suitability^1^, and volatile chemicals emitted by plants play central roles in this assessment^2^. Perception of a suitable habitat also stimulates reproductive behavior, including enhanced mating success and greater oviposition^3^,^4^.

In many insect species, especially nocturnal flying insects, sex pheromones are pivotal in intersexual communication. Sex pheromone blends are generally emitted by sexually receptive females that engage in a species-specific pattern of pheromone emission, termed calling behavior^5^. Conspecific males are exquisitely and specifically sensitive to their species-specific pheromone blend and they navigate upwind toward the calling females^6^. Both the emission of pheromone by females and male orientation to females are photoperiodically controlled. Moreover, sex pheromone production and calling behavior are generally under neuroendocrine regulation^7^,^8^.

Plant volatiles are often divided into several distinct categories: phenylpropanoids/ benzenoids, terpenoids and green-leaf volatiles (GLVs) that include C-6 aldehydes, esters and alcohols^9^,^10^, and they exert multi-factorial ecological effects on herbivores. Volatile compounds are usually emitted in small amounts by healthy plants, and herbivore or pathogen attack stimulates higher release rates. Thus, volatile compounds play pivotal roles in direct/constitutive and indirect plant defenses, but they can also affect insect reproductive behavior. Benzenoid and other volatile compounds in floral scent, which mediate pollination, are also involved in insect sexual attraction^11^,^12^. GLVs, which are widespread in angiosperm emissions, are widely known to enhance insect reproduction by improving males’ responses to sex pheromones^13^,^14^. Some insects only release sex pheromone when exposed to host volatile compounds^15^ and pheromone production can be stimulated by host volatile compounds^16^. In many insect species, host plant volatiles can synergize the effectiveness of sex pheromones^17^,^18^, and mechanistic neuroethological explanations for such effects have been proposed^19^,^20^. Importantly, antagonistic interactions between floral volatiles and male attraction to pheromone suggest a context-dependent interaction between these olfactory pathways^21^.

In contrast, non-host plant volatiles (NHPVs) are often repellents and/or deterrents for many insect species across several orders^22^,^23^,^24^. For example, *Aphis fabae* (Scop) seldom consume Brassicaceae, and they show behavioral avoidance to isothiocyanates, the volatile catabolites of glucosinolates that chemically characterize this plant family^22^. Other cases have focused on conifer-inhabiting beetles, which generally synthesize and release terpenoids as aggregation pheromones, but behaviorally avoid GLVs or C-8 bark volatiles that angiosperms emit^25^.

Few studies have explored the relationship between NHPVs and sex pheromone communication^2^. Generally, it has been hypothesized that detection of sex pheromone can be adversely affected in a “noisy” background and NHPVs can reduce the signal/noise ratio. For example, pheromone detection by olfactory receptor neurons and pheromone-guided orientation of *Spodoptera littoralis* can be disturbed by background odor, such as linalool, a plant terpene alcohol emitted from both host and non-host plants^26^,^27^. Likewise, the monoterpenoid citral reduces the attraction of male *Grapholita molesta* (Oriental fruit moth) to female sex pheromone^28^. We speculate that the relative dearth of research on NHPVs can be attributed to two reasons: First, while it is relatively easy to define “non-host”, especially for specialist herbivores, the universe of non-hosts is essentially boundless. Second, compounds considered to be NHPVs may in fact be found in small amounts and under unique conditions in host plants as well.

We hypothesize that some “novel” plant-derived compounds, which specialists rarely encounter within their habitat, could adversely affect their intersexual communication. We used the specialist *Plutella xylostella* as a model; it is globally distributed but feeds only on Brassicaceae crops. Calling females of this specialist release a multicomponent sex pheromone consisting of (*Z*)-11-hexadecenyl acetate, (*Z*)-11-hexadecenal and (*Z*)-11-hexadecen-1-ol to attract conspecific males^29^ and a mass ratio of 3:7:1 is most attractive to males in Chinese *P. xylostella* populations^30^. For NHPVs we chose turpentine (also called spirit of turpentine, oil of turpentine, wood turpentine), α-terpineol and eucalyptol. Turpentine is a distillate of pine resin composed mainly of the monoterpenes α-pinene and β-pinene, with lesser amounts of carene, camphene, dipentene and terpinolene. α-Terpineol was also identified as a major constituent of turpentine from a variety of *Pinus* species^31^ and *Cinamomum camphora* leaves^32^. Eucalyptol is a predominant compound in *Eucalyptus* species, accounting for >70% of the total VOC content in *Eucalyptus*^33^. Notably, all these terpenoids are rarely detected in vapor emissions of fresh leaves or inflorescences of the Brassicaceae^34^,^35^.

Although some NHPVs have been found to be repellents and/or deterrents for many insect species, the effects of the NHPVs on sex pheromone communication has not been tested systematically. In the present study, we assessed the effectiveness of intersexual communication with or without NHPVs. Firstly, electrophysiological (electroantennogram, EAG) tests were done to determine if the antennae of specialist herbivores are capable of receiving compounds that are not part of their host plant emissions. Secondly, host plant and non-host plant odors were established in laboratory context to compare calling behavior of females. Thirdly, the orientation of male adults to sex pheromone lures was tested with or without NHPV odors both in a wind tunnel and in open field. Moreover, the purpose of this study was to generate mechanistic results that might explain the low incidence of specialist herbivores in polyculture plant systems and to explore an innovative approach of NHPV-based mating disruption in moths.

## Results

### EAG Responses

**Figure 1:**
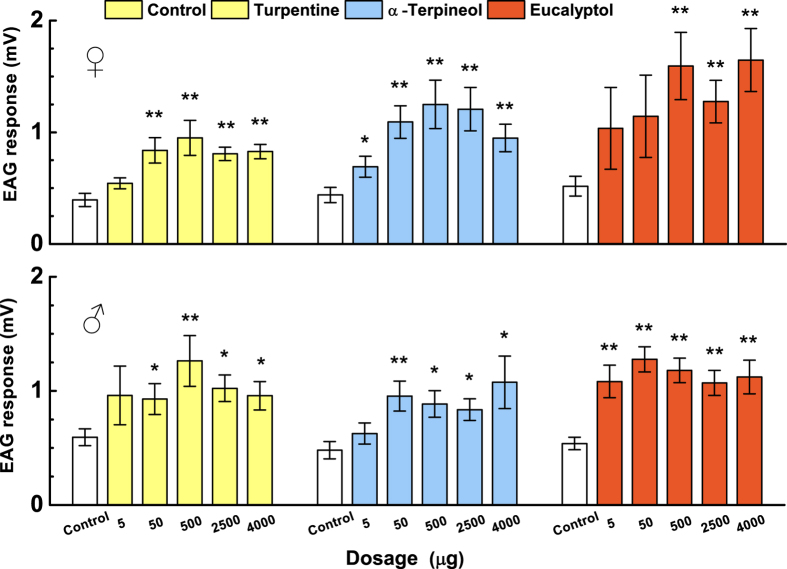
Electrophysiological responses of *P. xylostella* female and male antennae to NHPVs. Data are presented as mean values ± SEM (*N* = 8) and values from control and each dose were compared by Mann–Whitney *U* test (*P < 0.05, **P < 0.01).

Turpentine, α-terpineol and eucalyptol elicited significantly greater EAG responses from the antennae of both female and male *P. xylostella* than the paraffin control treatment (Fig. 1). Therefore, the antennae of male and female *P. xylostella* have receptors for these NHPVs. Notably, at the lowest dose tested (5 μg) sexual differences were also evident with female antennae being more sensitive to α-terpineol (*U* = 13.0, *P* = 0.046; males: *U* = 21.0, *P* = 0.248), whereas male antennae were more sensitive to eucalyptol (*U* = 1.5, *P* = 0.001; females: *U* = 23, *P* = 0.343).

### Calling Behavior of Females

**Figure 2:**
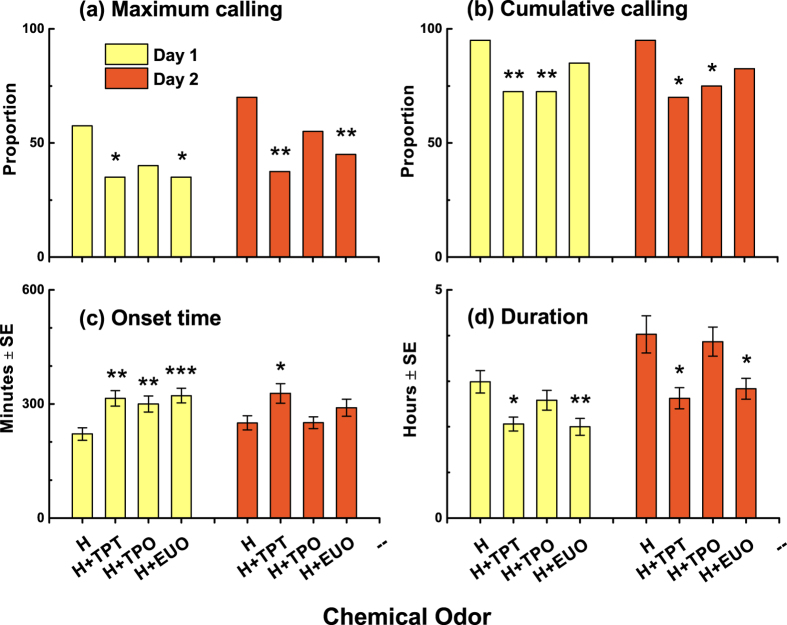
Calling behavior of female *P. xylostella* in the presence of plant odors. H, host odor; TPT, turpentine; TPO, α-terpineol; EUO, eucalyptol. Each terpenoid was loaded at a dose of 1000 μg onto a rubber septum. (**a**) Maximum calling denotes the largest number of females calling synchronously. (**b**) Cumulative calling is the total number of females that engaged in calling behavior. H and each H+terpenoid treatment in (**a,b**) were statistically compared using a χ^2^ test (*P < 0.05, **P < 0.01). (**c**) The onset time of calling is expressed as minutes after the onset of the observation at 18:00, and the scotophase was from 20:00 to 4:00 (8 h). (**d**) Duration is the hours that calling behavior lasted. Significant differences between H and each H+terpenoid treatment in (**c,d**) are indicated by asterisk (*P < 0.05; **P < 0.01; ***P < 0.001, Mann–Whitney *U* test).

Our observations of calling behavior support the hypothesis that NHPV terpenoids inhibited *P. xylostella* females from calling even in the presence of host odors (α = 0.05). The presence of turpentine (day 1: χ^2^ = 4.073, *P* = 0.044; day 2: χ^2^ = 8.498, *P* = 0.004) and eucalyptol (day 1: χ^2^ = 4.073, *P* = 0.044; day 2: χ^2^ = 5.115, *P* = 0.024) decreased the maximum calling proportion on two successive days (Fig. 2a). In addition, cumulative calling proportion was significantly reduced on two successive days in females exposed to turpentine (day 1: χ^2^ = 7.44, *P* = 0.006; day 2: χ^2^ = 8.658, *P* = 0.03) and α-terpineol (day 1: χ^2^ = 7.44, *P* = 0.006; day 2: χ^2^ = 6.275, *P* = 0.012), compared with females exposed to host plant alone (Fig. 2b).

Furthermore, of those females that called (day 1: host odor, *N* = 38; turpentine, *N* = 29; α-terpineol, *N* = 29; eucalyptol, *N* = 34; day 2: host odor, *N* = 38; turpentine, *N* = 28; α-terpineol, *N* = 30; eucalyptol, *N* = 33), the average onset time of calling was significantly delayed by NHPVs, but mainly on the first day of calling (turpentine: *U* = 289.5, *P* = 0.001; α-terpineol: *U* = 329.0, *P* = 0.005; eucalyptol: *U* = 315, *P* < 0.001). On the second day, females exposed to α-terpineol (*U* = 569.0, *P* = 0.99) and eucalyptol (*U* = 535.0, *P* = 0.287) showed no more differences in onset time of calling compared with females exposed to host odors alone (Fig. 2c). The results suggest that females adjust their responses to long-term exposure to NHPVs. Nevertheless, the average calling duration was significantly reduced over the 2-day experiment in the presence of turpentine (day 1: *U* = 357.0, *P* = 0.013; day 2: *U* = 361.5, *P* = 0.026) and eucalyptol (day 1: *U* = 412.5, *P* = 0.008; day 2: *U* = 454.5, *P* = 0.046), compared with females exposed only to host odors (Fig. 2d).

### Male Responses in the Wind Tunnel

**Figure 3:**
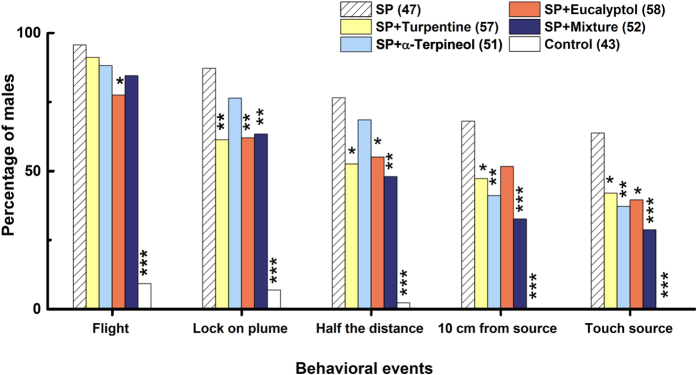
Behavioral responses of male *P. xylostella* in the wind tunnel. SP, ternary sex pheromone blend of (Z)-11-hexadecenyl acetate, (Z)-11-hexadecenal and (Z)-11-hexadecen-1-ol at 3:7:1 mass ratio. Mixture, ternary blend of turpentine, α-terpineol and eucalyptol at a 1:1:1 mass ratio. Numbers in parentheses indicate male replicates. Within each behavioral event, bars with asterisks above are significantly different from the SP bars (χ^2^ test, *P < 0.05; **P < 0.01, ***P < 0.001).

The sequential behavioral responses of *P. xylostella* males to odors in a wind tunnel varied significantly (Fig. 3). Of all males tested with sex pheromone lures alone, more than 63% landed on the mesh and searched the odor source. The proportion landing decreased significantly with the addition of turpentine (42.11%, χ^2^ = 4.87, *P* = 0.027), α-terpineol (37.25%, χ^2^ = 6.909, *P* = 0.009) and eucalyptol (39.66%, χ^2^ = 6.07, *P* = 0.014), and a mix of these 3 NHPVs at equal amounts (28.85%, χ^2^ = 12.186, *P* < 0.001).

Furthermore, most of the NHPV chemicals also inhibited early events in the sex pheromone orientation of males. For instance, turpentine and eucalyptol significantly reduced upwind navigational flight (χ^2^ = 8.736, *P* = 0.003). However, α-terpineol did not significantly inhibit the males’ response until they were within 10 cm of the source (χ^2^ = 7.132, *P* = 0.008).

### Field Trapping

**Figure 4:**
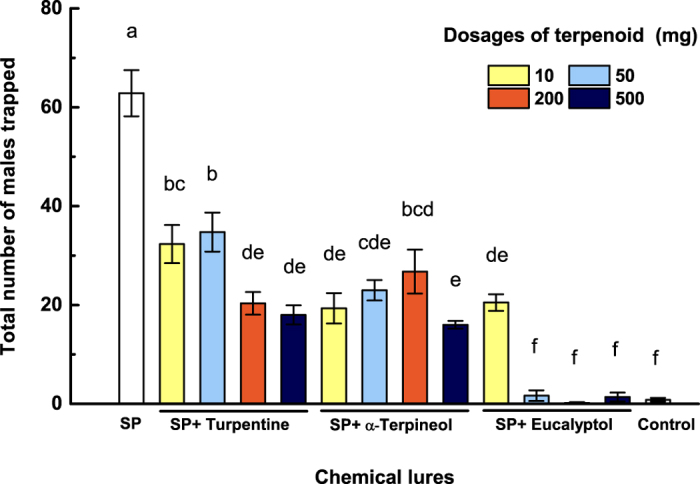
Attraction of male *P. xylostella* to different lure combinations in a *Brassica rapa Linnaeus* var. *glabra* Regel field in Shanghai, China. SP, ternary sex pheromone blend of (Z)-11-hexadecenyl acetate, (Z)-11-hexadecenal and (Z)-11-hexadecen-1-ol at 3:7:1 mass ratio. Each SP+terpenoid treatment had 6 replicates and SP-only lures had 8 replicates. Bars with the same letter are not significantly different [ANOVA on square root (*x* + 1); Duncan’s multiple range test, *P* ≥ 0.05].

The attractiveness of the ternary sex pheromone blend alone and in combination with NHPV terpenoids was compared in field assays. The results of the trapping experiment demonstrated that the presence of turpentine, α-terpineol or eucalyptol at all selected dosages significantly (*P* < 0.05) reduced captures of *P. xylostella* males in pheromone-baited traps (Fig. 4). However, the 3 terpenoids exerted differential effects on trap catch. Eucalyptol was especially effective at suppressing attraction to the sex pheromone, with dosages ≥50 mg (release rate of >0.89 mg/day, Supplementary Fig. S3) inhibiting male attraction to the level of control traps (Fig. 4).

## Discussion

Adults of both specialist and generalist herbivores can detect a wide range of plant odors and they even perceive some volatiles beyond the plant species they normally colonize. Electrophysiological detection of and behavioral modulation by constituents of non-host plant odors have been reported in conifer-inhabiting beetle species^25^ and in a few lepidopterans^2^,^36^. However, specific olfactory recognition of “strange” odorants by specialists has rarely been studied. Our results clearly demonstrate for the first time, that both adult females and males of the Brassicaceae-consuming specialist *Plutella xylostella* can also detect some non-host plant terpenoids seldom found in Brassicaceae, and these NHPVs inhibit adult reproductive behaviors.

Our results showed that the addition of turpentine to host odors consistently delayed the onset time of calling in females. Previous research has amply documented the capacity of females to respond to proximate environmental changes and modify their calling initiation and calling duration. For example, odors of *Brassica juncea* (Linnaeus) broadened the calling ages of female *P. xylostella* and advanced the onset of calling during the scotophase^37^. In our observations, female *P. xylostella* exposed to turpentine delayed calling, and eucalyptol and turpentine significantly reduced their calling durations. Presumably, by causing these shifts in calling onset and duration, NHPVs could limit the female’s mating opportunities.

Likewise, our behavioral tests in the wind tunnel and field trapping experiments indicated that NHPVs disrupt the male’s ability to navigate the sex pheromone plume. Nevertheless, not all NHPVs performed equally. In wind tunnel experiments, turpentine and α-terpineol appeared to exert their inhibitory effects at shorter distances than eucalyptol, whereas eucalyptol more efficiently inhibited males from approaching and touching the pheromone source. However, our field trials demonstrated that eucalyptol was much more effective at inhibiting attraction of males to sex pheromone. For example, when eucalyptol (ca. 0.89 mg/day, Supplementary Fig. S2) was used with sex pheromone, fewer males were trapped in pheromone traps than in control traps without pheromone. We suspect that the greater inhibitory performance of eucalyptol in the field than in the wind tunnel resulted from a combination of the close-range behavior of males (we used small 10 cm traps that would require very precise orientation), the formulations we used, and natural variations in field conditions.

Plant species diversity plays a vital role in buffering insect populations and modulating herbivore attack, especially in agricultural landscapes. Thus, forest and agricultural monocultures experience more frequent and devastating insect outbreaks than mixed-species forests and other natural ecosystems^38^,^39^. However, the mechanisms that underlie “association resistance”, whereby certain plants are protected by proximity to neighboring plant species, and for reduced pest abundance in polycultures, remain equivocal and not well understood. At the habitat or landscape level, suitable intercropping, “companion planting”, and plant rotation can limit herbivores from spreading^40^ or even interrupt their life cycles^41^. Moreover, greater plant species diversity also provides suitable alternative shelters^42^, microclimate, or food resources for predatory arthropods and parasitoids^43^.

Since olfactory orientation is the primary modality in habitat and host location by moths, pervasive NHPV odors are expected to interfere with host location via direct repellent or deterrent effects or by changing the signal-to-noise ratio in antennal olfactory processing, thus “camouflaging” biologically relevant odors. Two major hypotheses, the “masking hypothesis” and “resource concentration hypothesis”, have been proposed to describe non-host plant effects on host finding by herbivores^44^,^45^, and attempt to explain the observed patterns of pest outbreaks. Our electrophysiological and behavioral results demonstrated compound-specific effects of the NHPVs on pheromone responses of male diamondback moths, consistent with olfactory disruption. However, the effects of NHPVs on moth sexual communication (female calling behavior) suggest broader mechanisms, beyond masking or repellency. Turpentine, for example, influenced both the male’s orientation to sex pheromone and the female’s calling behavior, potentially lowering the probability of sexual encounters and successful mating. Thus, the effects of NHPVs in reducing herbivore attack in diverse plant communities might be explained, at least in part, by their suppression of moth sexual communication. The underlying sensory mechanisms for this interaction remain unknown. It is possible, however, that volatile terpenoids function as repellents. These compounds are often used in anthropogenic mixtures as mosquito repellents, and some birds provision their nests with aromatic leaves and branches to repel ectoparasites^46^.

Strategies of moth mating disruption are mainly based on the deployment of synthetic sex pheromones. Saturating the landscape with a high concentration of sex pheromone leads to false trail following, plume competition, disorientation, and therefore a decline in successful mating^47^. Pheromone formulations with incomplete blends or with “off-ratios” also have potential for disrupting mating by “unbalancing” olfactory processing in males^48^,^49^. Female calling behaviors are also modulated by sex pheromone reception^50^,^51^, a form of autodetection that may mediate interaction among females to avoid competition. In general, mating disruption with synthetic chemicals may be costly, and its effect on female behavior may be limited^52^.

Our results demonstrate that terpenoids, representing NHPV chemicals, are detected by the antennae of both females and males; they disrupt female calling and diminish the male’s behavioral responses to sex pheromone. Therefore, these NHPVs can interfere with sexual activity and mating success. We propose that NHPVs have potential in mating disruption in field applications, alone or in combination with synthetic sex pheromones. However, much more research is needed to understand the mechanisms of NHPV action and their effectiveness in the field.

## Methods

### Insects

Large numbers of male and female larvae and pupae of *Plutella xylostella* were collected in a Chinese cabbage (*Brassica rapa* Linnaeus var. *glabra* Regel) field, at Lin’an city (30.3 °N, 119.7 °E), Hangzhou, China. These insects were not exposed to pesticides both before and after their collection from the field. Insects were reared on cabbage seedlings in large plastic-screen cages (3.5 × 3.5 × 1.5 m) maintained at 25 ± 2 °C and 70 - 75% relative humidity under a photoperiod of 16L: 8D. Pupae were removed to another cage (0.5 × 0.5 × 1 m) and newly emerged adults were provided with cotton pads impregnated with ca. 5% hydromel (honey in water). After the 3rd generation, pupae were separately transferred into test tubes and kept under the same ambient conditions.

### Sex pheromone and non-host plant terpenoids

The three synthetic sex pheromone components of *P. xylostella*, (Z)-11-hexadecenyl acetate, (Z)-11-hexadecenal and (Z)-11-hexadecen-1-ol, were obtained from Shin-Etsu Chemical Co. Ltd. All pheromone chemicals were confirmed to be >97% by GC. Commercial turpentine (99% by GC, CAS 8006-64-2), α-terpineol (98% by GC, CAS 98-55-5), and eucalyptol (98% by GC, CAS 470-82-6) were all purchased from Chinese Medical Chemical Company.

### EAG recordings

We adopted and slightly modified a classical electroantennogram (EAG) method^14^ to test antennal responses to plant chemicals. Adult females, 2 days post eclosion, were transferred into the experimental conditions for acclimatizing. An antenna from *P. xylostella* was excised at the base and mounted with conducting gel onto a ground electrode; 2 distal segments were also removed and the distal end of the antenna was connected to the recording electrode. Individual terpenoids in paraffin oil were applied to strips of filter paper (2 cm × 0.5 cm) that served as odor sources. Filter paper treated only with 5 μL of paraffin oil was used as control. The filter paper was inserted into a Pasteur pipette, and the tip of the pipette was inserted into a small hole (3 mm diameter) of a main airflow tube (12 mm diameter) in which a continuous, clean humidified airflow (240 mL/min) was blown onto the antenna. When stimulating, a 0.5 s puff of odorized air (2 mL at 240 mL/min) was introduced through the pipette, transporting the odorants to the antenna, using an electronically controlled stimulus controller (CS-55, Syntech, the Netherlands). The EAG signals were amplified and processed with a PC-based signal acquisition controller (IDAC-2, Syntech). A stimulation sequence with one antenna consisted of a control puff followed by increasing dosages of non-host plant odors.

### Observation of calling behavior

Each two-day old adult female was placed into a clear acrylic tube (OD, 30 mm; height, 95 mm), together with a sugar water-impregnated cotton ball, and restricted to the distal half of the tube by a screen. In the other part of each tube, a fresh Chinese cabbage (*Brassica rapa* Linnaeus var. *glabra* Regel) seedling (height, 80 ± 5 mm) and a natural red rubber septum (ID, 9 mm; Institute of Plant Physiology & Ecology, Shanghai Institutes for Biological Sciences, Chinese Academy of Sciences) impregnated with 1000 μg (100 μg/μL in hexane) of terpenoid chemical (estimated release rates in Supplementary Fig. S1) were both inserted serving as host and non-host odor source, respectively (Supplementary Fig. S2). A fresh plant seedling with a blank septum acted as host odor only control.

Experiments were carried out in an experimental chamber at 25 °C, 70 ± 5% RH and a photoperiod of 16L: 8D (L-off 20:00). We observed females at 30-min intervals for two consecutive days with each observation period lasting 11 hours (18:00–5:00), from 2 hours before the onset of the scotophase to 1 hour after the onset of the photophase. The host seedlings and natural rubber septum were all renewed after the first day observations. All observations were under red light (25 watt incandescent). Females were considered calling when the abdomen was extended and the ovipositor extended without oviposition behavior. Calling parameters included (a) the maximum calling proportion at all times, (b) the cumulative calling proportion, (c) the average onset time of calling and (d) duration of calling.

### Wind tunnel assays

The cylindrical wind tunnel apparatus (diameter, 50 cm; length, 180 cm), constructed with Plexiglas, was placed in an environmental chamber (27 ± 2 °C, 70–80% RH). The odor-impregnated filter paper lure (4 cm × 1.5 cm) was contained in a dark mesh cage (30 cm from the upwind end of the tunnel, 25 cm height) to avoid visual cues. Males were released 30 cm from the downwind end of the tunnel. Thus, the complete flight path from the release unit to the odor lure was 120 cm. Air speed in the wind tunnel was set to ca. 40 cm/s using visible smoke. Male moths were introduced into the wind tunnel at least 1 hour prior to the assays for acclimation.

A ternary blend of (Z)-11-hexadecenyl acetate, (Z)-11-hexadecenal and (Z)-11-hexadecen-1-ol in a 3:7:1 ratio was diluted in hexane to a concentration of 0.1 μg/μL and each terpenoid was diluted in hexane to a concentration of 1 μg/μL. 100 μL of sex pheromone and terpenoid solution were transferred to two separate filter papers (ca. 0.5 cm apart) and the hexane was allowed to evaporate. Redistilled hexane alone was applied to control filter papers. For each replicate, one moth was released and its behavioral response was observed for up to 5 min. Each treatment was replicated >40 times with separate male moths. Filter papers with sex pheromone were replaced with new filter papers after 10 replicates and NHPV-treated papers were renewed after 3 replicates. The internal surface of the wind tunnel was wiped with hexane after each treatment. The odor-guided upwind flight of males was divided into five sequential events^14^: taking flight, locking on the plume, oriented flight for half the distance to the source, 10 cm from source and touching the source.

### Field trapping of males

To investigate whether NHPV terpenoids influence pheromone-guided flight of males in a crucifer field, we conducted field experiments in a Chinese cabbage (*Brassica rapa* Linnaeus var. *glabra* Regel) field in Shanghai, China (June 12–17, 2013). Each rubber septum was impregnated with 10 μg of (Z)-11-hexadecenyl acetate, (Z)-11-hexadecenal and (Z)-11-hexadecen-1-ol at 3:7:1 mass ratio. In the field experiment, cotton balls were placed in 1.5-mL polyethylene centrifuge tubes with a 2-mm-diameter hole in the lid. The cotton balls were moistened with a series of dosages of NHPVs which released at different rates (Supplementary Fig. S2).

Pheromone lures and NHPV chemical dispensers were tied together with a metal wire. Basin traps (10 cm diameter) with 5% soap in water were hung ~20 cm above the plant canopy. Traps were placed ~6 m apart, with treatments randomly interspersed. Insects captured in the traps were counted and removed daily. After each inspection, the traps were re-randomized in order to avoid an effect of trap location.

### Statistical analyses

For EAG analysis, peak amplitudes elicited by NHPV terpenoids and the paraffin control were compared using Mann–Whitney *U* test. For “maximum calling rate” and “cumulative calling rate” analyses, numbers of calling or non-calling females were weighted, and the distributions of control and the non-host-treated group were compared through a 2 × 2 contingency table using a χ^2^ test. Parameters of onset calling time and calling durations in non-host treatment and control were also compared by Mann–Whitney *U* test. The distribution of insect responses to different odor sources from the wind tunnel bioassay was analyzed using a χ^2^ test. In the field trapping experiments, trap catch was subjected to a square root transformation (*x* + 1) to stabilize the variance. Trap catches were then compared by one-way analysis of variance (ANOVA) followed by Duncan’s multiple range test (α = 0.05).

## Additional Information

**How to cite this article**: Wang, F. *et al*. Non-Host Plant Volatiles Disrupt Sex Pheromone Communication in a Specialist Herbivore. *Sci. Rep.* **6**, 32666; doi: 10.1038/srep32666 (2016).

## Supplementary Material

Supplementary Information
