# Supplementary material for: Non-Host Plant Volatiles Disrupt Sex Pheromone Communication in a Specialist Herbivore
Source: Sci Rep. 2016 Sep 2;6:32666. doi: 10.1038/srep32666 (PMC5009357; doi:10.1038/srep32666)
Supplement: Supplementary Information [file srep32666-s1.pdf]

Supplementary information

Non-Host Plant Volatiles Disrupt Sex Pheromone Communication in a Specialist Herbivore

Fumin Wang[1], Jianyu Deng[1,*], Coby Schal[2,*], Yonggen Lou[3], Guoxin Zhou[1], Bingbing Ye[1], Xiaohui Yin[1], Zhihong Xu[1], Lize Shen[1]

[1]Department of Plant Protection, School of Agriculture and Food Science, Zhejiang Agriculture and Forestry University, Lin'an, Hangzhou, Zhejiang, 311300, China;

[2]Department of Entomology and W. M. Keck Center for Behavioral Biology, North Carolina State University, Campus Box 7613, Raleigh, NC 27695–7613, USA;

[3]National Key Laboratory of Rice Biology, Institute of Insect Science, Zhejiang University, Hangzhou, Zhejiang, 310058, China;

*To whom correspondence should be addressed.

E-mail: jydeng70@aliyun.com ; coby@ncsu.edu

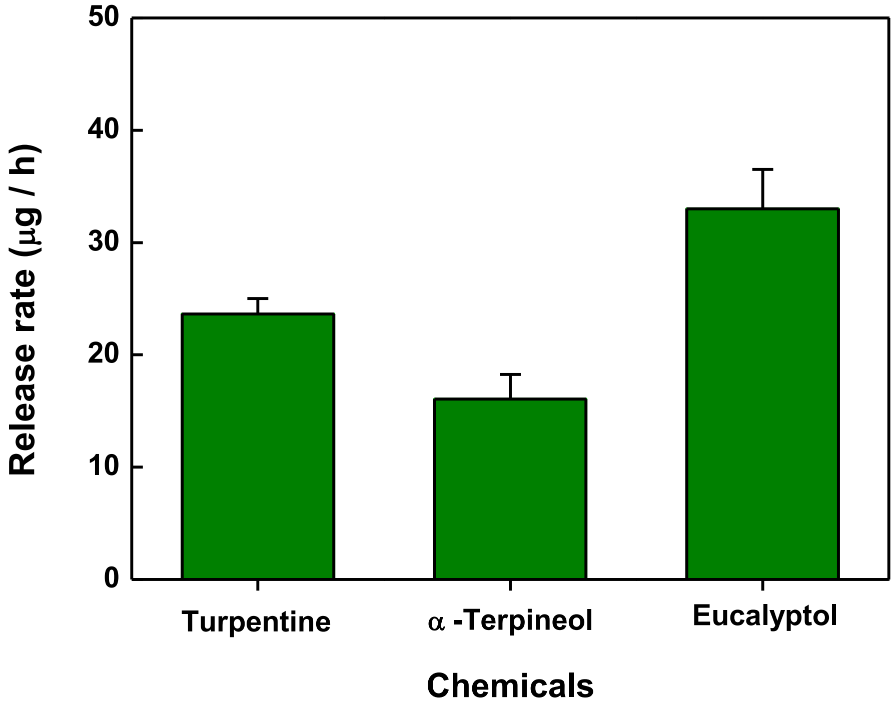

Figure S1 Mean estimated release rates + SEM (*N* = 30) of NHPVs loaded at 1000 µg per rubber septum used in observations of calling behavior. Groups of 10 rubber septa were weighed together on an electronic balance (Mettler-Toledo, d = 0.0001 g). Release rates were calculated as mass loss of the rubber septa throughout the 11-h observation period (18:00 – 5:00).

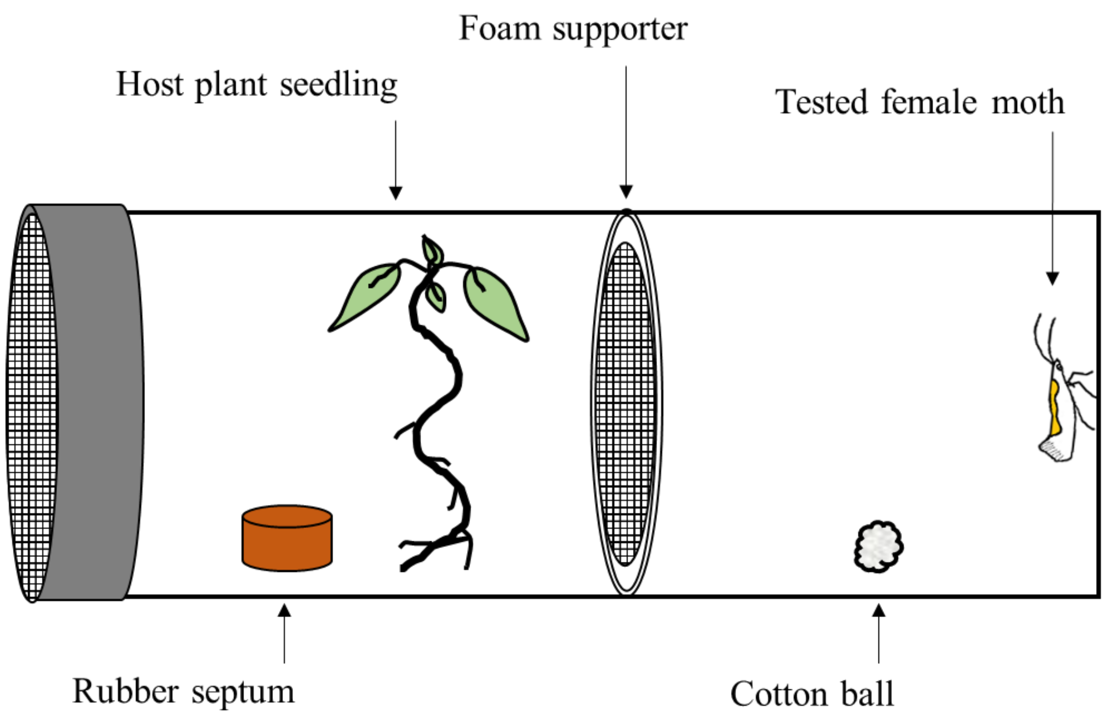

Fig S2 Schematic diagram of arena utilized in observation of calling behavior of female diamondback moth. Odor sources and insect were compartmentalized by window screening supported by a foam loop.

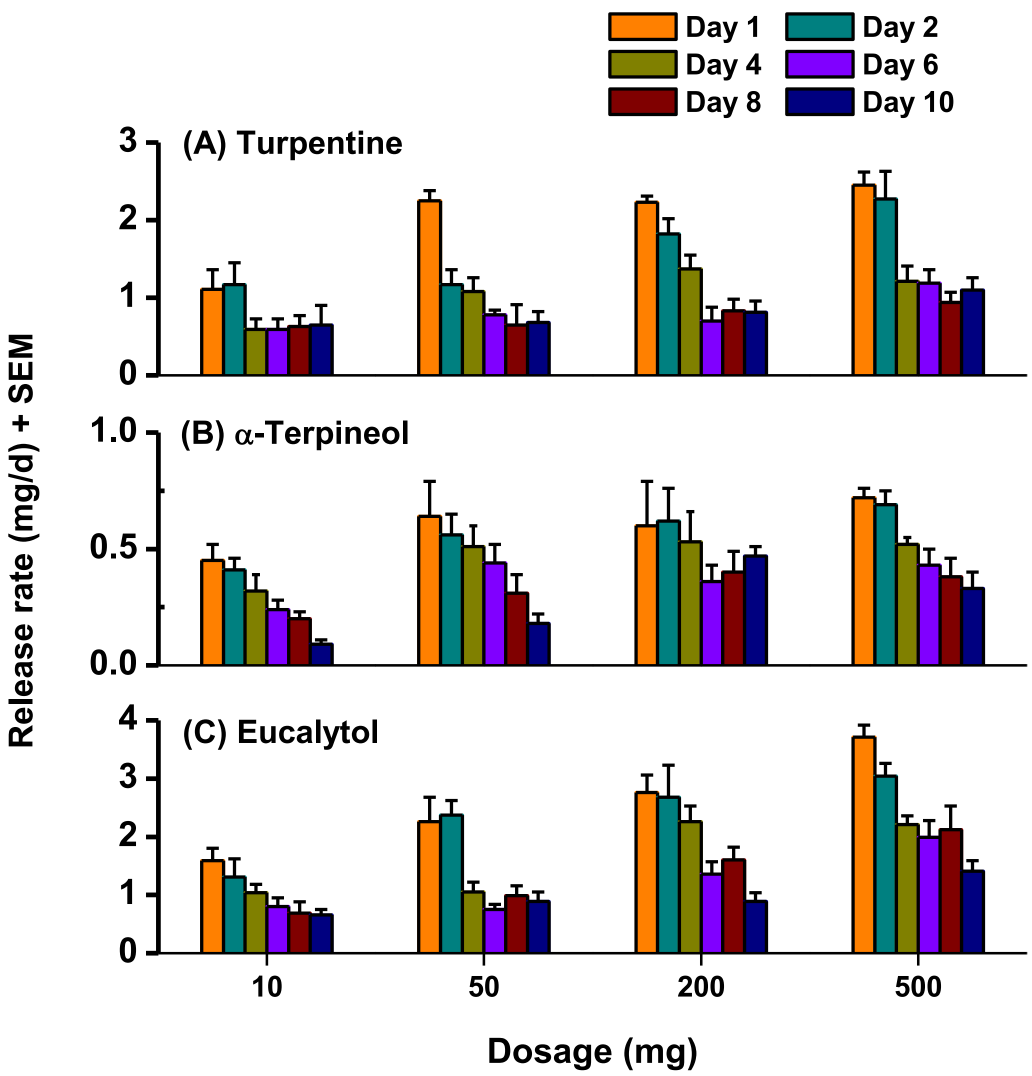

Figure S3 Mean estimated release rates + SEM (n = 5) of NHPVs in a Chinese cabbage (*Brassica rapa Linnaeus* var. *glabra* Regel) field. Release rates were calculated by the decreasing weights of polyethylene centrifuge tubes over consecutive days.
